# Sarcopenic Obesity as a Risk Factor for Cardiovascular Disease: An Underrecognized Clinical Entity

**DOI:** 10.17925/HI.2023.17.2.6

**Published:** 2023-12-01

**Authors:** Aditya John Binu, Nitin Kapoor, Saptarshi Bhattacharya, Kamal Kishor, Sanjay Kalra

**Affiliations:** 1. Department of Cardiology, Christian Medical College, Vellore, India; 2. Department of Endocrinology, Diabetes and Metabolism, Christian Medical College, Vellore, India; 3. Non-communicable Disease Unit, Baker Heart and Diabetes Institute, Melbourne, Victoria, Australia; 4. Department of Endocrinology, Indraprastha Apollo Hospital, New Delhi, Delhi, India; 5. Department of Cardiology, Rama Hospital, Karnal, India; 6. Department of Endocrinology, Bharti Hospital, Karnal, India; 7. University Center for Research & Development, Chandigarh University, Mohali, India

**Keywords:** Aging, lifestyle, muscle, obesity, sarcopenia, sarcopenic obesity

## Abstract

Sarcopenic obesity (SO) is a chronic condition and an emerging health challenge, in view of the growing elderly population and the obesity epidemic. Due to a lack of awareness among treating doctors and the non-specific nauture of the associated symptoms, SO remains grossly underdiagnosed. There is no consensus yet on a standard definition or diagnostic criteria for SO, which limits the estimation of the global prevalence of this condition. It has been linked to numerous metabolic derangements, cardiovascular disease (CVD) and mortality. The treatment of SO is multimodal and requires expertise across multiple specialties. While dietary modifications and exercise regimens have shown a potential therapeutic benefit, there is currently no proven pharmacological management for SO. However, numerous drugs and the role of bariatric surgery are still under trial, and have great scope for further research. This article covers the available literature regarding the definition, diagnostic criteria, and prevalence of SO, with available evidence linking it to CVD, metabolic disease and mortality, and an overview of current directives on management.

With the global increase in life expectancy, the proportion of elderly people in the community is expected to increase gradually. This is referred to as the ‘Coming of the Gray Dawn’ or the ‘Gray Wave’, and highlights the need for current day healthcare professionals to better understand the specific disease processes that occur in the elderly.^1,2^ One such key change that occurs with increasing age is the alteration in body composition. With each passing year, the body experiences a gradual reduction in muscle mass (sarcopenia) and a relative increase in fat mass (obesity) (*Figure 1*).^3–5^ Though this change occurs in all individuals, it is a modifiable phenotype and depends on the dietary intake and exercise performance of an aging individual.^6,7^ There is emerging literature on the impact of sarcopenic obesity (SO) on different cardiometabolic risk factors, but a comprehensive review on considering it as a risk factor for cardiovascular disease (CVD) is needed.

In this manuscript, the authors have attempted to bridge this gap by comprehensively reviewing the available literature regarding the definition, diagnostic criteria, and prevalence of SO, along with the available evidence linking it to CVD, metabolic disease and mortality, and also provide an overview of the current management directives. This will help to better understand the importance of recognising, evaluating and treating SO, to reduce cardiovascular (CV) mortality. Furthermore, the authors have also provided future directions for research on this subject to help identify possible research questions that are currently not addressed in the available literature.

## Bidirectional impact

A reduction in muscle mass limits a given individuals' physical activity and mobility, contributing to obesity.^4^ But an increase in fat mass also compromises muscle mass – adipose tissue can either physically decrease muscle quality by fat infiltration or by its systemic impact of increasing insulin resistance and pro-inflammatory markers (*Figure 2*).^8^ This leads to a vicious cycle, thus further enhancing progression of declining muscle mass and increasing adiposity at a rapid pace,^9^ especially in the elderly. This bidirectional impact also explains why these two disorders often co-exist rather than occurring in isolation.

## Definition of sarcopenia and sarcopenic obesity

The term ‘sarcopenic obesity’ was first coined by Baumgartner et al.^10^ SO has been defined by the European Society for Clinical Nutrition and Metabolism (ESPEN) and the European Association for the Study of Obesity (EASO) as a clinical and functional condition characterized by the coexistence of excess fat mass (high body fat percentage) and sarcopenia (low skeletal muscle mass accompanied by low muscle function).^11,12^ Sarcopenia is diagnosed based on two or three of the following parameters (varying between different working groups): (a) low muscle mass, (b) low muscle strength, and (c) low physical performance.^13–15^ The perceived prevalence of sarcopenia is lower when all three criteria are utilised, but higher when any two criteria are satisfied.^16^ Sarcopenia may also be defined as the total or appendicular skeletal muscle mass (ASM) in weight/height^2^ (kg/m^2^) of an individual being below two standard deviations of a reference population of the same gender.^17^ The prevalence of sarcopenia is higher in women than in men.^18^ Obesity may be defined as a body mass index (BMI) >30 kg/m^2^,^19^ or a high total or percentage fat mass as assessed by dual-energy X-ray absorptiometry (DXA), bioelectrical impedance analysis or by using other parameters such as visceral fat area or waist circumference (WC). The perceived prevalence of SO in a population aged ≥60 years ranged from 4.4% to 84.0% in men and from 3.6% to 94.0% in women, in a study done between 1999-2004 using the National Health and Nutrition Examination Surveys database in the United States, which may be attributed to the diversity of diagnostic criteria.^20^

**Figure 1:**
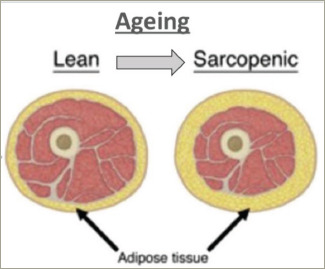
Change in body composition with aging

## Current gaps in the sarcopenia literature

SO may be associated with significant frailty, disability, and morbidity, as we will see in the latter part of this manuscript. Current diagnostic criteria for SO are heterogenous, ill-defined and do not allow for appropriate patient identification and stratification, in their present form. A 19-26-fold variation in sex-specific rates was noted on reviewing eight definitions for SO.^20^ As can be inferred from the wide variability in the prevalence of SO assessed in various studies, and which highlights the compelling need for further research to ensure uniform diagnostic criteria and management guidelines.

Despite an increase in the knowledge of the mediators and metabolic pathways involved in the pathogenesis of SO, gross lacunae still persist.^21^ Areas of potential research include investigations into the mediators of the positive effects of exercise, the role of gut hormonal systems and microbiota metabolism in SO, and the interaction of nutritional regulation and skeletal muscle homeostasis.^6,22,23^ These research outcomes would help in the prevention and management of patients with SO.

Areas with scope for further research could also help develop dietary, prevention and treatment protocols for optimising skeletal heath in subjects with obesity. A growing body of evidence lends support for increased protein and amino acid intake, as well as skeletal muscle anabolism with maintenance of lean body mass. A protein intake of 1.0–1.2 g per kg of ideal body weight per day has been suggested as recommended intake in healthy geriatric populations.^24–26^ In addition, exercise training, especially resistance exercise, has been shown to be effective in improving muscle function and mass.^27^

## Unique South Asian phenotype

In India, there is a very high prevalence of individuals who are overweight and obese, and an even higher prevalence of sarcopenia. The overall prevalence of SO in India was noted to be 8.7% in the Longitudinal Aging Study in India.^9,28^ The chief predictors of SO were noted to be higher age, urban residence, a geographical location in western or southern India, consumption of tobacco or alcohol, lack of physical activity, ophthalmological comorbidities and the presence of diabetes.^9^ The prevalence of SO in a north Indian cohort of community-dwelling healthy adults as per the Sarcopenic Obesity-Chandigarh Urban Bone Epidemiological Study (SO-CUBES) was determined to be 5.4–6.3% utilizing BMI, WC and DXA-derived fat mass (FM) percentage.^29^ In this study, apparently healthy individuals aged ≥ 20 years with no prior history of any co-morbidities were recruited from the community and underwent body composition analysis, dominant handgrip strength (HGS), and usual gait speed (GS) to assess sarcopenia. However, the younger age is probabaly the reason behind a relatively lower prevalence in this cohort.Asian Indians have been noted to have a small body size with centripetal obesity, as characterised by a high waist-to-hip ratio, WC, visceral fat, and posterior subcutaneous abdominal fat.^30,31^ This phenotype has been termed as ‘normal weight obesity’ (normal BMI associated with a high percentage of body fat) and has been noted to have an independent association with increased CV mortality compared to individuals with a normal body fat percentage.^32^ Muscle strength and mass is lower in South Asians than Caucasians. The South Asian community has also been noted to be ethnically and phenotypically different from Eastern and South Eastern Asian nations.^9,27^ In view of these differences, international guidelines need to be adapted for the Asian and South Asian community with caution.^33^

**Figure 2:**
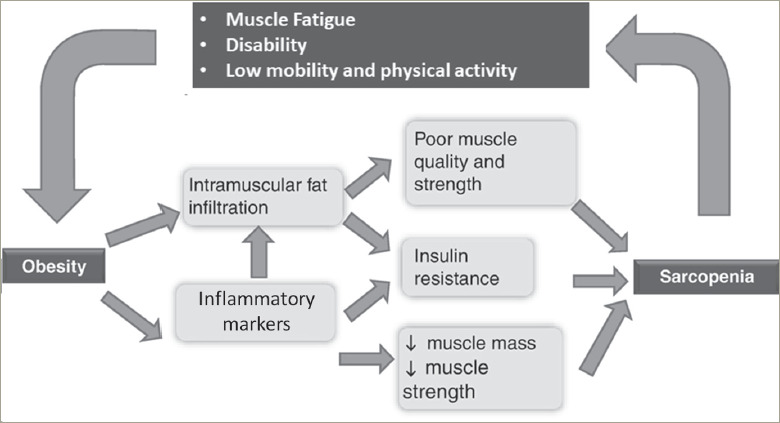
The bidirectional impact of sarcopenia and obesity

**Table 1:** Measurement cut-offs for sarcopenia and obesity from various studies^34–38^

Author (year)	Sarcopenia measure	Sarcopenia measurement (cut-off value)	Obesity indicator	Study population
Baumgartner (2004)^34^	ASM/height^2^	DXA (male <7.26 kg/m^2^, female <5.45 kg/m^2^)	Body fat ( male ≥28%, female ≥40%)	New Mexico Elder Health Survey
Villareal (2005)^35^	ASM/height^2^	ASM (<5.45 kg/m^2^, sex not specified)	BMI (≥30 kg/m^2^)	Young healthy population
Bouchard (2009)^36^	ASM/height^2^	DXA (male <8.51 kg/m^2^, female <6.29 kg/m^2^)	Body fat ( male ≥28%, female ≥35%)	Nutrition as a Determinant of Successful Aging Study
Kim (2009)^37^	ASM/height^2^	DXA (male <7.40 kg/m^2^, female <5.14 kg/m^2^)	Body fat ( male ≥20.2%, female ≥31.7%)	Korean Sarcopenic Obesity Study
Levine (2012)^38^	(ASM x 100)/ (body mass)	DXA (male <25.7%, female <19.4%)	WC (male <102 cm, female <88 cm)	National Health and Nutrition Examination Survey

*ASM = appendicular skeletal muscle; BMI = body mass index; DXA = dual-e nergy X-ray absorptiometry; WC = waist circumference.*

## Diagnosis of sarcopenic obesity

SO has various diagnostic criteria, as denoted in *Table 1*.^34–38^ Various diagnostic tests are employed in the diagnosis of sarcopenia, including HGS, GS, whole-body DXA, short physical performance battery, squad-j ump, countermovement jump, and 10 m and 20 m sprint performance.^39,40^ Anthropometric indices such as BMI, WC, waist-to-height ratio (WHtR), weight-adjusted waist index (WWI), along with newer indices as demonstrated in *Table 2*, may also be employed in screening for SO.^41,42^ Higher WWI, WHtR and WC quartiles were associated with higher risk of SO.^41^ A systematic review in 2019 revealed that 19 different measurements of sarcopenia and 10 measurements of adiposity had been applied across various studies; of these, ASM divided by weight (ASM/wt.) or adjusted by height in meters squared (ASM/h^2^) and BMI were, the most commonly applied measurements of sarcopenia and obesity, respectively. The heterogeneity of the diagnostic assessment was further exacerbated by the application of varying cut-offs for the same measurements.^11^ Hence, a consensus on diagnostic criteria for SO, followed by validation based on homogeneous studies and databases, is the need of the hour as this will ensure an assessment of the correct prevalence, evaluation and treatment of SO across different ethnicities and countries.

## Impact of sarcopenic obesity on cardiovascular risk factors

### Sarcopenic obesity and diabetes

SO has been demonstrated to have an association with hyperglycaemia and insulin resistance in numerous studies. In a cross-sectional study conducted in 2020, in Singapore with 1,235 patients with type 2 diabetes, SO was detected in >20% of patients with type 2 diabetes mellitus aged more than 45 years of age.^43^ Similarly, subjects with SO were observed to have the highest risk of insulin resistance and dysglycaemia in a cross-sectional analysis of 14,528 adults from the NHANES III cohort.^44^ In the Korean Sarcopenic Obesity Study (KSOS), which included 810 subjects (414 patients with diabetes and 396 control subjects) who were examined using DXA, SO was noted to be associated with insulin resistance, inflammation, and vitamin D deficiency.^45^ Furthermore, SO was associated with insulin resistance, metabolic syndrome, dyslipidemia, and vitamin D deficiency in a cross-sectional analysis of 2,943 participants aged 60 years or above from the Korea National Health and Nutrition Examination Survey (KNHANES).^46^ Another Korean study reported a higher risk of diabetes in patients with SO (odds ratio [OR]: 2.16, 95% confidence interval [CI]: 1.08–3.27) than in the sarcopenia group (OR: 1.24, 95% CI: 0.86–2.15).^47^

### Sarcopenic obesity and hypertension

A cross-sectional study of older adults from the KNHANES database noted that SO had a greater association with increased risk of hypertension.^48^ The risk of hypertension was higher in the sarcopenia (OR: 2.48, 95% CI: 1.89–6.16), obesity (OR: 3.15, 95% CI: 2.76–3.59), and SO (OR: 6.42, 95% CI: 4.85–8.48) groups than in the non-sarcopenia, non-obesity group.^48,49^ The 10-year risk of hypertension was found to be higher in patients with SO than in those with sarcopenia alone or those who did not have sarcopenia or obesity, but similar to those with obesity alone.^50^ Studies have also demonstrated that the prevalence of SO is much higher in patients with chronic diseases such as hypertension, diabetes, and dyslipidaemia.^51^

**Table 2:** Various anthropometric indices for sarcopenic obesity

Waist-calf circumference ratio Waist circumference/calf circumference	Normal <2.5 Suspected SO 2.5–3.5 Confirmed SO >3.5
Calf central index Calf circumference/waist–hip ratio	Normal >30 Suspected SO 30–40 Confirmed SO <40
Body-calf index BMI/calf circumference	Normal <0.6 Suspected SO 0.6–1.0 Confirmed SO >1.0

*BMI = body mass index; SO = sarcopenic obesity.*

### Sarcopenic obesity and dyslipidaemia

SO has been noted to have a significant association with dyslipidaemia.^52^ Studies from the KHNANES cohort have demonstrated that male patients with SO had a higher risk of dyslipidaemia (OR: 2.82, 95% CI: 1.76–4.51) compared with those with obesity alone (OR: 2.12, 95% CI: 1.11–4.07) or sarcopenia alone (OR: 1.46, 95% CI: 1.01–2.11).^52^

### Sarcopenic obesity and metabolic syndrome

SO has been found to be significantly associated with metabolic syndrome. A greater risk of metabolic syndrome was noted by Lim et al. among adults with SO (OR: 8.28, 95% CI: 4.45–15.40) than among those with obesity (OR: 5.51, 95% CI: 2.81–10.80) or sarcopenia (OR: 2.64, 95% CI: 1.08– 6.44) in a cross-sectional study of 565 patients aged ≥65 years from the Korean Longitudinal Study on Health and Aging (KLoSA).^47^ Another study conducted among 600 older adults from Taiwan showed that the group with SO had a higher risk of metabolic syndrome (OR: 11.59, 95% CI: 6.72–19.98) than the group with obesity alone (OR: 7.53, 95% CI: 4.01–14.14) or sarcopenia alone (OR: 1.98, 95% CI: 1.25–3.16).^53^

### Sarcopenic obesity and osteoporosis

A study conducted across 3,385 males and 4,064 females from the KHNANES cohort demonstrated that the risk of osteoporosis was higher in the SO group (OR: 8.67, 95% CI: 4.19–17.94 in men; OR: 2.93, 95% CI: 1.99–4.32 in women).^54^ Another cross-sectional study using data from 2,893 subjects from the KNHANES database showed a greater risk of knee osteoarthritis in the SO (OR: 3.51, 95% CI: 2.15–5.75) and non-SO (OR: 2.38, 95% CI: 1.80–3.15) groups, but not in the sarcopenic, nonobesity group.^55^ Yet another study reported lower bone mineral density and a higher risk of non-vertebral fracture in older adults with SO than in those without sarcopenia, without obesity, and with only obesity.^56^

### Sarcopenic obesity and coronary artery disease

A cross-sectional analysis of Korean adults from the KNHANES database assessing the association between the 10-year CVD risk (estimated using the Framingham risk score) and SO revealed a higher 10-year CVD risk in the SO group compared with the non-sarcopenia non-obesity group (OR: 2.49, 95% CI: 1.53–4.06 in men; OR: 1.87; 95% CI: 1.02–3.41 in women), even though the risk of CVD was not high in the groups with sarcopenia or obesity alone.^57^ However, contrasting reports were seen in a prospective cohort study of older men from the British Regional Heart Study.^57^ In this study, no significant increase in the risk of coronary heart disease (fatal or non-fatal myocardial infarction) or CVD events (non-fatal myocardial infarction, non-fatal stroke, or fatal CVD) were noted in men with SO.^57^ Numerous cross-sectional studies have also reported that older adults with SO did not have a significantly higher prevalence of CVD than those without sarcopenia or obesity.^58,59^

**Table 3:** Clinical implications of sarcopenic obesity on cardiometabolic health*

1	SO is grossly underdiagnosed due to lack of awareness and criteria
2	SO is associated with increased risks of disability, mortality, metabolic diseases and cardiovascular diseases compared with individuals with obesity or sarcopenia alone
3	A combination of aerobic and resistance exercises is helpful to address both obesity and sarcopenia, respectively
4	A daily protein intake of 1.2–1.6 g/kg/day has been proposed in patients with SO; caution is to be exercised in patients with renal impairment
5	The utility of anti-obesity medications in the management of SO is not well studied
6	Many novel drug targets (such as tesamorelin [a growth-hormone-releasing hormone analog], myostatin antibodies [LY2495655, bimagrumab, REGN1033, PF-06252616, BMS-986089, PINTA-745] and recombinant irisin) are currently in different stages of drug development

*Clinical implications are based on the authors experience in this area SO = sarcopenic obesity.

### Sarcopenic obesity and mortality

Various studies have shown an association between SO and increased mortality risk. In the British Regional Heart Study, an analysis of 4,107 men aged 60–79 years revealed that the risk of all-cause mortality was increased in men with SO.^53^ Subjects with a high WC (>102 cm) and in the lowest quartile of midarm muscle circumference had a 55% increase in mortality risk compared with non-sarcopenic, non-obese men over 6 years of follow up.^60^ On further follow up of the cohort after 11 years, there was a 72% increased risk of mortality in men with SO compared with the non-sarcopenic, non-obese group after adjustment for lifestyle and CV risk factors.^61^ A prospective analysis of 4,652 participants aged ≥60 years from the NHANES III cohort showed higher risk of allcause mortality in women with SO (HR: 1.29, 95% CI: 1.03–1.60) than in women without sarcopenia or obesity over a 14-year follow-up period.^62^ A meta-analysis of 12 prospective cohort studies found that the risk of mortality was highest in patients with SO (HR 1.24, 95% CI 1.12–1.37) compared with healthy individuals.^63^

### Clinical implications

With a steady increase in the global geriatric population and the concurrent obesity epidemic, SO is a rapidly burgeoning health phenomenon. As sarcopenia and obesity share pathological factors including aging, hormones, and immunological factors, SO has a greater cumulative risk of negative health outcomes when compared to sarcopenia or obesity alone. In the absence of a global consensus definition and diagnostic criteria, SO is probably underdiagnosed as a clinical entity. As we may note from the aforementioned evidence, SO is associated with an increased risk of disability, mortality, metabolic diseases, CVD, and other comorbidities, compared with sarcopenia or obesity alone. In terms of assessing the overall health burden of SO, we are probably witnessing only the tip of the iceberg. The various clinical implications have been summarized in *Table 3* and are now discussed in more detail.

### Sarcopenic obesity and exercise

There is a relative scarcity of data with regard to the management of SO. A regimen of weight loss without exercise leads to a simultaneous reduction of both fat mass and lean mass, which further aggravates sarcopenia. In a systematic review conducted by Weinheimer EM et al., it was reported that the addition of exercise to energy restriction can attenuate the loss of lean mass, despite not appearing to have any significant additive effect on weight reduction.^22^ A fat-free mass (FFM)>15% was observed in approximately 81% of overweight/obese individuals following energy restriction alone compared with 39% using energy restriction and exercise, respectively.^22^ This suggests that exercise is an effective tool to help preserve FFM after moderate energy restriction induced weight loss, which is important for combating SO.

In view of the possible benefits of exercise in countering SO, prescribing a good exercise regimen is essential.^64^ Evidence indicates that a combination of aerobic and resistance exercise is the best possible regimen.^65^ Resistance training has been shown to be more useful against sarcopenia, while aerobic exercise has a better efficacy against obesity. Hence, it could be concluded that their combination could provides the best results in SO, although evidence is currently lacking.

A randomized controlled trial conducted in older adults with obesity demonstrated an improvement in the physical performance test which was greater in the combination exercise group than in the resistance-only or aerobic-only groups (29%, 14%, and 14%, respectively) over 6 months of follow up. The decrease in lean mass was smaller in the combination and resistance groups than in the aerobic group (3%, 2%, and 5%, respectively).^66^

A few trials have compared resistance exercise with no exercise.^67,68^ Resistance exercise has been shown to increase lean mass and physical capacity in older female subjects with SO.^67,68^

Periodization, a systematic variation in the specificity of physical training, intensity and volume, has also emerged as a potential strategy to improve muscle performance.^69^

### Sarcopenic obesity and diet

With regard to dietary regimens in patients with SO, there is evidence to suggest that adequate protein intake is essential in SO.^70^ Protein supplementation in addition to resistance exercise was shown to reduce fat mass and increase lean mass, upper body strength, and leg strength compared with resistance exercise alone.^70^ A daily protein intake of 1.2–1.6 g/kg/day has been proposed in patients with SO; caution is to be exercised in patients with renal impairment.^71^ Limited evidence exists to suggest that a dietary supplementation with vitamin D, amino-acid supplementation, tea catechins, and combination exercise (aerobic plus resistance) for 3 months can improve body fat mass and physical function, but not muscle mass, in elderly women with SO.^72^

### Sarcopenic obesity and anti-obesity medications

There is a relative paucity of trials on the utility of conventional antiobesity medications (orlistat, phentermine, lorcaserin, liraglutide etc.) on SO.^73^ The rapidity of weight reduction following the use of anti-obesity medications may cause rapid simultaneous loss of muscle mass, though this is has not yet been confirmed in SO by any published studies. Drugs such as tesamorelin (a growth hormone-r eleasing hormone analog), myostatin antibodies (LY2495655, bimagrumab, REGN1033, PF-06252616, BMS-986089, PINTA-745) and recombinant irisin have been shown to have beneficial effects in patients with SO.^74–77^

### Sarcopenic obesity and bariatric surgery

There is limited evidence on the effects of bariatric surgery in patients with SO.^78^ A risk of excessive loss of muscle mass is present in the event of weight loss, and if surgery is not followed by regular exercise.^78^ A prospective cohort trial comparing the effect of gastric bypass and sleeve gastrectomy in patients with SO and non-SO showed that despite baseline differences in muscle mass between both groups, there was no difference between the groups 12 months after bariatric surgery.^79^ This indicates that patients with sarcopenia do not lose more muscle mass despite similar weight loss. However, more randomized trials are required before recommending bariatric surgery in patients with SO.

## Summary

SO is a chronic condition and an emerging health challenge, connected to the confluence of the rise in the elderly population and the obesity epidemic. The disparity in diagnostic criteria was a disadvantage faced by previous trials on SO, when defining inclusion criteria for subjects in a clinical trial; this has been resolved to an extent by the ESPEN/EASO consensus on definition and diagnostic criteria, published in 2022.^1^ Screening for SO in the elderly population should be prioritized to permit early diagnosis of SO and early initiation of treatment in order to minimize unfavourable outcomes and improve quality of life. As this review has highlighted, SO is associated with a vast burden of metabolic disorders, morbidity, risk of CVD and mortality. Therapeutic regimens are in the nascent stages of development and need a multi-specialty and multimodality approach to tackle this challenge with changes in diet, exercise regimens, pharmacological management and rarely, surgical intervention. This review also stresses the need for an increased awareness of SO, including amongst medical specialties beyond geriatrics. A broader recognition is crucial for a comprehensive understanding of the true health burden of SO, as it will enable us to discern, for example, the incidence of SO under different conditions, such as malignancies and other medical conditions.
